# Green extraction of chitin from *Litopenaeus vannamei* shell waste using deep eutectic solvents coupled with ultrasound-generated bulk nanobubbles

**DOI:** 10.1016/j.ultsonch.2026.107979

**Published:** 2026-07-27

**Authors:** Azadeh Ghiasvand, Masoud Rezaei, Shahab Naghdi

**Affiliations:** Seafood Processing Department, Marine Sciences Faculty, Tarbiat Modares University, Noor, Iran

**Keywords:** Chitin, *Litopenaeus vannamei*, Deep eutectic solvent (DES), Ultrasound-assisted nanobubbles, Green extraction

## Abstract

A novel green strategy for chitin extraction from *Litopenaeus vannamei* shell waste was developed using choline chloride (ChCl)-based deep eutectic solvents (DESs) coupled with nanobubbles. Initially, three DES systems, including ChCl-citric acid (ChCl-CA), ChCl-lactic acid (ChCl-LA), and ChCl-glycerol (ChCl-Gly), were evaluated for chitin extraction performance. Among them, ChCl-Gly exhibited the highest extraction yield (39.85%), significantly outperforming ChCl-CA (26.53%), ChCl-LA (28.45%), and the conventional chemical method (17.91%) (P < 0.05). Therefore, ChCl-Gly was selected for further nanobubble-assisted extraction experiments at ultrasonic powers ranging from 100 to 400 W. The incorporation of bulk nanobubbles significantly enhanced extraction efficiency compared with pristine DES treatment. The maximum extraction yield (41.40%) was achieved by the ChCl-Gly-NB 100 W treatment, whereas ChCl-Gly-NB 400 W provided the best overall performance, with high extraction yield (39.49%), deproteinization efficiency (>96%), demineralization efficiency (>86%), while preserving the characteristic α-chitin structure. The extracted chitin exhibited high crystallinity (77.32%), favorable thermal stability (Tmax = 336 °C), and a porous fibrillar morphology, indicating effective removal of non-chitinous components. Overall, the synergistic DES-BN system offers a sustainable, low-toxicity, and highly efficient alternative to conventional acid–alkali extraction methods for shrimp shell valorization and green chitin production.

## Graphical abstract

**Figure:**
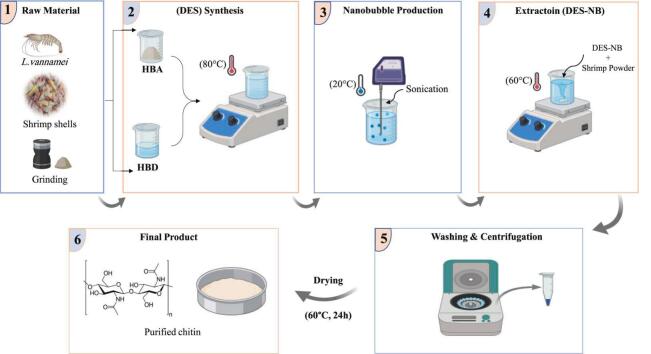

## 1 Introduction

The rapid expansion of global aquaculture has led to a critical environmental challenge regarding waste management, with Pacific white shrimp (*Litopenaeus vannamei*) processing alone discarding 50–60% of raw material weight as shells, heads, and tails [1]. Conventional disposal practices pose severe threats to aquatic ecosystems [2]. However, these marine by-products represent a rich source of high-value bioactive compounds, particularly chitin (20–30%), which holds immense potential for food, pharmaceutical, and biomaterial applications [3]. Consequently, the efficient valorization of this biopolymer not only mitigates ecological pollution but also enhances the economic sustainability of the seafood sector [4]. Chitin is a linear polysaccharide composed of β-(1 → 4) linked 2-acetamido-2-deoxy-d-glucopyranose (GlcNAc) monosaccharide repeating units characterized by a highly crystalline structure, which limits its direct solubility and industrial application [5]. It is frequently converted into chitosan, its deacetylated derivative, which is widely utilized in drug delivery, tissue engineering, and active packaging systems due to its superior biocompatibility and non-toxicity [6], [7]. Traditional chemical extraction of chitin relies on successive demineralization and deproteinization stages using concentrated acids and strong bases, which induces severe polymer degradation and generates hazardous, corrosive chemical effluents [8]. Although biological alternatives (enzymatic or microbial) are eco-friendly, they suffer from prolonged processing cycles and high cost-associated constraints [9]. Deep eutectic solvents (DESs) are a new class of green solvents formed by combining a hydrogen bond acceptor (HBA) and a hydrogen bond donor (HBD), resulting in a eutectic mixture with a melting point lower than that of its individual components [10], [11]. Owing to their negligible vapor pressure, low volatility, ease of preparation, low cost, biodegradability, low toxicity, tunable physicochemical properties, and potential for recovery and reuse, DESs have emerged as environmentally friendly alternatives to conventional organic solvents. Recent studies have demonstrated that choline chloride-based DESs can be successfully recycled and reused over multiple extraction cycles while maintaining high extraction efficiency, highlighting their potential for sustainable and large-scale industrial applications [12], [13]. Consequently, DESs have attracted considerable attention as sustainable extraction media for the recovery of bioactive compounds and biopolymers, including chitin [14]. Beyond these advantages, DES systems enable a streamlined, one-pot process where demineralization and deproteinization occur simultaneously, contrasting with the cumbersome stepwise sequence of chemical extractions [15]. To further accelerate mass transfer and improve DES efficiency, auxiliary physical treatments such as ultrasound, microwave or conventional heating have been investigated [14], [16], [17]. Recently, nanobubble (NB) technology (gas bubbles < 500 nm) has gained attention as a novel physical processing aid [18]. Due to their high stability, elevated internal pressure, and large surface area, NBs enhance interfacial contact and induce localized nanoconfinement effects upon collapse, thereby creating micro/nanopores to boost extraction yields without damaging the polymer backbone [19]. Despite the growing body of literature on DES-mediated extraction, the synergistic integration of nanobubble technology as a physical intensifier to optimize this green process remains completely unexplored. Therefore, this study evaluates the feasibility of a novel green extraction platform for isolating chitin from *L. vannamei* shell waste using various choline chloride-based DESs coupled with nanobubbles. The ultimate objective is to systematically investigate the effects of this combined system on extraction yield, purity, and the physicochemical properties of the recovered chitin, establishing a direct comparison with the conventional chemical isolation method.

## 2 Materials and methods

### 2.1 Materials

Shell waste of whiteleg shrimp, *Litopenaeus vannamei*, was obtained from a local seafood processing company. Choline chloride (≥99%), citric acid (≥99%), lactic acid (85–90%), glycerol (≥99%), acetic acid, sodium hydroxide (NaOH), and hydrochloric acid (HCl) were purchased from Merck, and other used materials were analytical grade.

### 2.2 Preparation of shrimp shell powder

Shrimp shells were thoroughly washed with distilled water and dried in a hot-air oven at 60 °C for 48 h. The dried shells were subsequently ground and sieved through a 60-mesh screen [20]. The obtained shrimp shell powder was stored at − 23 °C until further use.

### 2.3 Preparation of deep eutectic solvents (DESs)

Three DES systems were prepared using choline chloride as the hydrogen bond acceptor (HBA) and citric acid, lactic acid, or glycerol as hydrogen bond donors (HBDs). Choline chloride was mixed with citric acid or lactic acid at a molar ratio of 1:1.5, while the molar ratio of choline chloride to glycerol was 1:2 (Table 1). The mixtures were heated under continuous magnetic stirring at 90 °C until transparent and homogeneous liquids were obtained, according to the method described by [21].

**Table 1:** Composition and molar ratios of prepared DESs.

**Hydrogen bond acceptor** (**HBA**)	**Hydrogen bond donor** (**HBD**)	**Molar ratio** (**HBA:HBD**)
Choline chloride	Glycerol	1:02
Choline chloride	Lactic acid	01:01.5
Choline chloride	Citric acid	01:01.5

### 2.4 Chitin extraction using DES

Shrimp shell powder was added to each DES at a solid-to-solvent ratio of 1:20 (w/w). The mixtures were magnetically stirred at 100 °C for 3 h. Subsequently, 7.5% (v/v) acetic acid was added to the glycerol-based DES sample, followed by an additional treatment at 100 °C for 1 h. After cooling to room temperature, the suspensions were washed repeatedly with distilled water and centrifuged until neutral pH was achieved. The recovered solids were then dried in an oven at 60 °C [21].

### 2.5 Generation of nanobubbles in DES by ultrasonication process

Evaluation of the chitin extraction yields revealed that the ClCh-Gly-based deep eutectic solvent (DES) outperformed both ChCl-LA and ChCl-CA treatments. Accordingly, this highest-yielding DES system was designated as the optimal treatment for both chitin extraction and nanobubble-assisted processing. Bulk nanobubbles were generated in the choline chloride/glycerol DES using ultrasonic cavitation according to the method reported by [22]. Ultrasonic treatments were performed at power levels of 100, 200, 300, and 400 W for 10 min. During sonication, the temperature of the DES was maintained at 20 °C using an ice bath.

### 2.6 Chitin extraction using nanobubble-enriched DES (DES–NB)

Shrimp shell powder was dispersed in the nanobubble-enriched DESs (namely ChCl-Gly-NB 100 W, 200 W, 300 W, and 400 W) and was subsequently stirred at 60 °C for 3 h. Thereafter, 7.5% (v/v) acetic acid was added, and the mixture was further treated at 100 °C for 1 h. The resulting suspension was washed with distilled water and centrifuged repeatedly until neutral pH was reached. The extracted chitin was subsequently dried at 60 °C [21], [22].

### 2.7 Conventional chemical extraction of chitin

Chitin was also extracted using a conventional two-step chemical method. Demineralization was first carried out using 1 M HCl at a solid-to-liquid ratio of 1:10 (w/v) at 60 °C for 3 h. The demineralized residue was then subjected to deproteinization using 1 M NaOH at a ratio of 1:10 (w/v) at 90 °C for 3 h. After each treatment step, the samples were washed thoroughly with distilled water and centrifuged until neutral pH was attained, followed by drying at 60 °C [23].

### 2.8 Determination of extraction yield and removal efficiencies

The chitin extraction yield was calculated as the percentage ratio of the dry weight of extracted chitin to the initial dry weight of shrimp shell powder. Demineralization efficiency was determined by ash analysis after combustion of the samples in a muffle furnace at 525 °C and calculating the reduction in mineral content. Deproteinization efficiency was evaluated using the Bradford assay by measuring absorbance at 595 nm and determining the reduction in protein content relative to the untreated control [16].

### 2.9 Fourier transform infrared spectroscopy (FTIR)

FTIR spectra of the extracted chitin samples were recorded using the KBr pellet method. Briefly, a small amount of chitin was thoroughly mixed and finely ground with KBr powder using a mortar and pestle. The mixture was then compressed into homogeneous pellets. Spectral analyses were performed using a Tensor II FTIR spectrometer (BRUKER, Germany) over the wavenumber range of 4000–400 cm⁻^1^.

Transmittance data were converted to absorbance values using the equation (1):

(1) A = 2 − log(%T)

The degree of deacetylation (DD) and degree of acetylation (DA) were calculated according to the following equation (2):

(2) *DA* (%) = (*A*_1655_/*A*_3450_) × 100

### 2.10 X-ray diffraction analysis (XRD)

X-ray diffraction patterns of the chitin samples were obtained using an X-ray diffractometer (X'Pert3). Measurements were conducted over a 2θ range of 5–60° at a scanning rate of 5° min⁻1. The crystallinity index (CrI) was calculated using the Segal method [21] according to the following equation (3):

(3) CrI (%) = ((I110-Iam)/I110) * 100

### 2.11 Thermogravimetric analysis (TGA/DTG)

Thermogravimetric analysis (TGA) and derivative thermogravimetry (DTG) were performed using a STA300 thermal analyzer (Hitachi, Japan). Samples were heated from 30 to 1000 °C at a heating rate of 10 °C min⁻^1^ under a nitrogen atmosphere [24].

### 2.12 Scanning electron microscopy (SEM)

The surface morphology of the chitin samples was examined using a Phenom ProX scanning electron microscope. Prior to imaging, the samples were sputter-coated with a thin layer of gold under vacuum conditions to improve electrical conductivity. SEM micrographs were recorded at an accelerating voltage of 15 kV [24].

### 2.13 Statistical analysis

All experiments were performed in triplicate (n = 3). Statistical analyses were conducted using one-way analysis of variance (ANOVA), followed by Duncan post hoc test at a significance level of p < 0.05. Data analysis was performed using SPSS software (version 26).

## 3 Results and discussion

### 3.1 Proximate composition analysis

The proximate composition of *L. vannamei* shell powder used in the present study is presented in Table 2. The obtained values were consistent with those previously reported by [25]. Minor variations in shell composition may be attributed to differences in shrimp species, geographical and aquaculture conditions, harvesting season, and processing methods, as previously suggested by [26].

**Table 2:** Proximate composition of *L. vannamei* shell powder.

**Protein** (**%**)	**Lipid** (**%**)	**Moisture** (**%**)	**Ash** (**%**)	**crude fiber**
38.93 ± 1.78	9.43 ± 1.01	69.67 ± 0.89	15.51 ± 1.32	33.85 ± 2.38

All experiments were performed in triplicate (n = 3).

### 3.2 Extraction yield

The extraction yields of chitin obtained from shrimp shell waste using different deep eutectic solvents (DESs), including ChCl-LA, ChCl-CA, and ChCl-Gly, were significantly different from that obtained by the conventional chemical method (Chem) (Table 3). Among all treatments, ChCl-Gly exhibited the highest extraction yield (39.85%), which was substantially higher than that of the conventional chemical extraction method (17.91%) (p < 0.05). This finding is in agreement with [16], who reported that DES-based extraction systems provide higher chitin recovery than conventional acid–alkali treatments. Similarly, [27] reported a chitin yield of approximately 29.20% using choline chloride-lactic acid DES, which is relatively comparable to the ChCl-LA and ChCl-CA systems obtained in the present study. The extraction performance of deep eutectic solvents (DESs) depends on the combined effects of their physicochemical properties, including the hydrogen-bonding network, viscosity, polarity, and molecular interactions with the target matrix [28]. The superior extraction yield obtained with ChCl–Gly can be attributed to its favorable physicochemical properties, particularly its lower viscosity, well-developed hydrogen-bonding network, and improved penetration into the shrimp shell matrix, which enhance mass transfer and facilitate impurity removal. In contrast, the acidic nature of ChCl–LA and ChCl–CA may alter their interactions with shell components, thereby affecting chitin recovery under the extraction conditions [29]. Therefore, the higher extraction yield of ChCl–Gly is likely due to a balanced combination of efficient impurity removal and better preservation of the chitin structure [27]. Collectively, these findings demonstrate the strong potential of ChCl-Gly as an environmentally friendly and efficient alternative to conventional chemical extraction methods for chitin recovery from shrimp shell waste. Based on the obtained results, ChCl-Gly was selected for subsequent nanobubble-assisted extraction experiments due to its superior extraction yield and favorable physicochemical properties. Although no significant differences were observed between ChCl-Gly and ChCl-LA in terms of deproteinization and demineralization efficiencies, the higher extraction yield of ChCl-Gly justified its selection for further processing. Moreover, previous studies have demonstrated that nanobubble concentration and stability decrease considerably under acidic conditions, accompanied by an increase in bubble size [30]. Therefore, selecting a DES system with suitable physicochemical properties capable of stabilizing nanobubbles was essential. Accordingly, the ChCl-Gly DES was selected for nanobubble generation at ultrasonic powers of 100, 200, 300, and 400 W. As shown in Table 3, the extraction yield of chitin was significantly influenced by ultrasonic power during nanobubble generation. The ChCl-Gly-NB 100 W treatment exhibited the highest extraction yield (41.40%), whereas the lowest yield was observed for ChCl-Gly-NB 400 W (39.49%). Higher ultrasonic powers produced smaller bulk nanobubbles with a larger specific surface area, thereby enhancing mass transfer and facilitating the removal of proteins and minerals from the shrimp shell matrix [31]. Consequently, the more efficient removal of these non-chitinous components slightly reduced the recovered dry mass, resulting in a lower apparent extraction yield. Therefore, the slight decrease in extraction yield reflects enhanced chitin purification rather than degradation of the chitin structure, as further supported by the increase in the degree of acetylation (DA) [32]. As confirmed by the deproteinization results presented in Table 3. Overall, nanobubble-assisted DES extraction significantly improved chitin recovery compared with the DES system without nanobubbles. Similar positive effects of nanobubbles on the extraction of bioactive compounds have also been reported by [33].

**Table 3:** Extraction yield, deproteinization, demineralization, crystallinity index, and degree of acetylation of extracted chitin samples.

**Treatment**	**Extraction yield** (**%**)	**Dm** (**%**)	**Dp** (**%**)	**Crl** (**%**)	**DA** (**%**)
**ChCl-CA**	26.53 ± 0.54 ^D^	89.02 ± 0.25^B^	94.51 ± 0.46^B^	85.49	71.2
**ChCl-LA**	28.45 ± 2.44^C^	88.14 ± 0.36^B^	94.32 ± 0.35^B^	84.62	85.1
**ChCl-Gly**	39.85 ± 0.23^c.AB^	88.71 ± 0.46^B^	93.76 ± 0.81^b.B^	72.2	80.1
**ChCl-Gly − NB 100 W**	41.40 ± 0.41 ^a.A^	81.70 ± 0.24 ^e.E^	94.23 ± 0.61^b.B^	79.85	72.4
**ChCl-Gly − NB 200 W**	40.66 ± 0.58 ^ab.AB^	81.28 ± 0.65 ^e.E^	94.98 ± 0.48 ^ab.AB^	82.1	74.8
**ChCl-Gly − NB 300 W**	40.23 ± 0.08 ^bc.AB^	85.29 ± 0.52 ^d.D^	96.67 ± 1.35 ^a.A^	81.59	78.6
**ChCl-Gly − NB 400 W**	39.49 ± 0.04^c.B^	86.94 ± 0.57^c.C^	96.58 ± 027 ^a.A^	77.32	83.2
**Chem**	17.91 ± 0.71 ^d.E^	91.80 ± 0.47 ^a.A^	96.67 ± 1.63 ^a.A^	91.3	65.4

**Note:** Uppercase letters indicate significant differences among the means of treatments in the first and second groups (DES and DES-NB), whereas lowercase letters indicate significant differences among the means of treatments within the second group (DES-NB).

### 3.3 Deproteinization efficiency

The deproteinization (DP) efficiency of chitin extracted using ChCl-Gly DES combined with nanobubbles generated at different ultrasonic powers is presented in Table 3. A significant increase in protein removal efficiency was observed with increasing ultrasonic power. Among the DES-based treatments, ChCl-Gly-NB 300 W and ChCl-Gly-NB 400 W exhibited the highest deproteinization efficiencies, reaching 97.97% and 97.91%, respectively. These values were comparable to the conventional chemical treatment (97.97%) and, in some replicates, even exceeded it. [34] similarly reported deproteinization efficiencies above 95% using comparable DES systems, supporting the findings of the present study. Furthermore, the deproteinization efficiency obtained by the chemical method was consistent with the results reported by [23]. Compared with the study of [35], who reported a DP value of 76.34%, the significantly higher protein removal observed in the present work may be attributed to the incorporation of nanobubbles into the extraction system. The enhanced deproteinization efficiency can be associated with the increased generation of nanobubbles at higher ultrasonic powers [36]. Upon collapse, nanobubbles generate nanojets and localized mechanical stresses that facilitate disruption of the shell matrix and promote the release of proteins from cellular structures, thereby improving deproteinization efficiency [37]. Altogether, the combined application of nanobubble technology and ChCl-Gly DES demonstrated excellent deproteinization performance for chitin extraction from shrimp shell waste. At higher ultrasonic powers, the DP values approached 98%, comparable to conventional chemical extraction. In addition to high extraction efficiency, DES systems offer environmental advantages such as biodegradability, low toxicity, and solvent recyclability, highlighting their potential as green alternatives to conventional extraction methods [35].

### 3.4 Demineralization efficiency

Despite the absence of strong acids and the relatively mild extraction temperature (60 °C), the demineralization (DM) efficiency increased progressively with increasing ultrasonic power during nanobubble generation. Among the DES-NB treatments, ChCl-Gly-NB 400 W exhibited the highest mineral removal efficiency (90.68%), which was significantly higher than that of ChCl-Gly-NB 100 W (86.90%) (Table 3). Although the chemical treatment achieved the highest DM value (94.15%), the difference between the chemical method and ChCl-Gly-NB 400 W was relatively small (∼3.5%). The improved demineralization performance can also be attributed to nanojet formation following nanobubble collapse. These nanojets facilitate disruption of interactions between mineral components and the chitin matrix, thereby enhancing mineral removal efficiency [37]. Nevertheless, the superior demineralization performance of the chemical method highlights the dominant role of strong acids such as HCl in calcium carbonate removal. Considering that DESs are biodegradable, recyclable, and environmentally benign alternatives to strong mineral acids and alkalis, the obtained demineralization efficiencies can still be regarded as highly satisfactory [15]. Moreover, the DM values obtained by the conventional chemical method were consistent with those reported by [23].

### 3.5 FTIR analysis

**Fig. 1:**
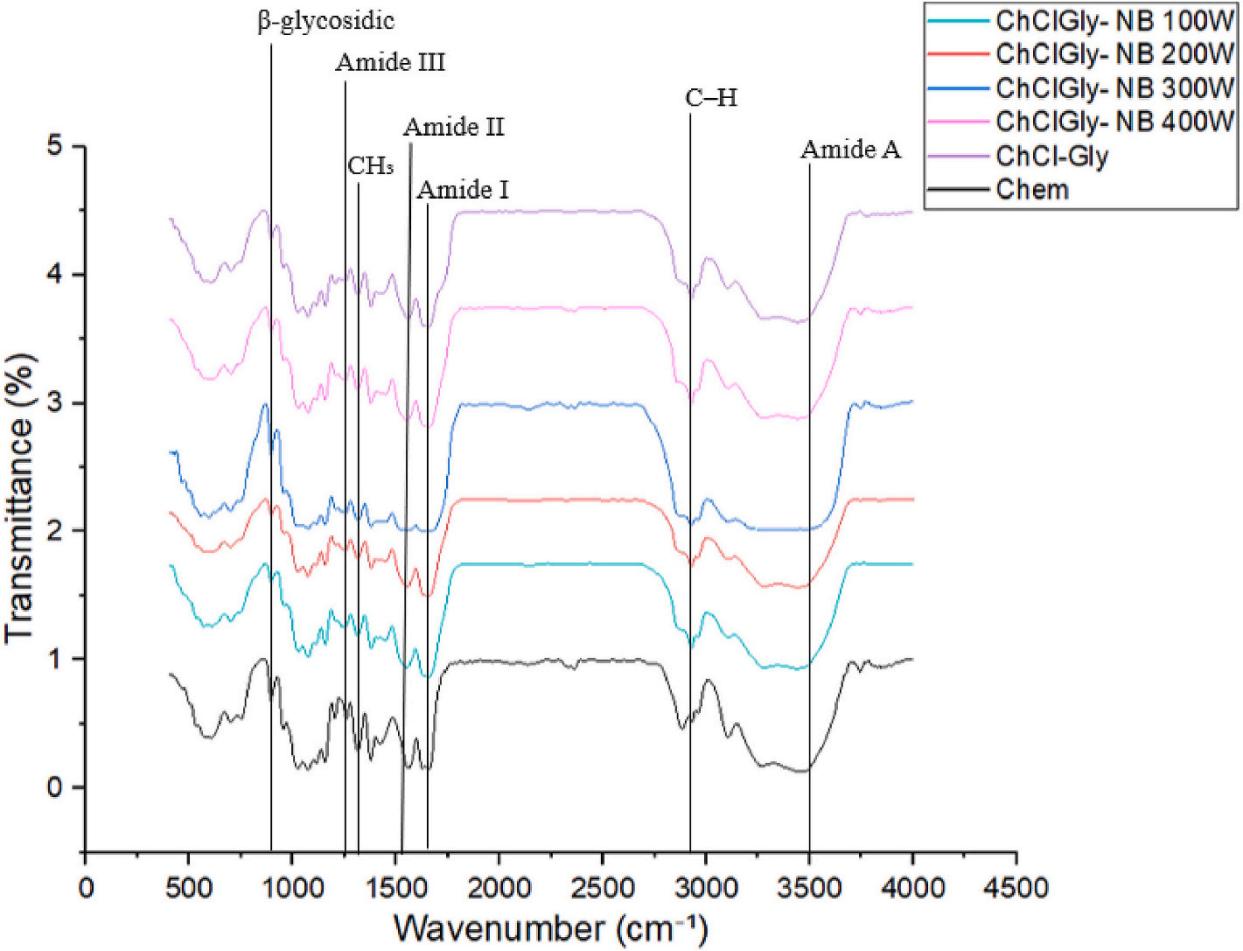
The FTIR spectra of all extracted chitin samples.

The FTIR spectra of all extracted chitin samples were acquired over a wavenumber range of 4000 to 400 cm^−1^; the corresponding spectral profiles are illustrated in Fig. 1 and Table 4. Overall, all samples exhibited characteristic absorption bands consistent with standard α-chitin spectra reported in the literature. Although slight shifts in peak positions and variations in peak intensity were observed among some samples, the presence of all characteristic functional groups confirmed successful chitin extraction. In the ChCl-Gly-NB 100 W sample, the Amide II band appeared at 1530 cm⁻^1^, slightly lower than the typical reference range (1550–1560 cm⁻^1^). This shift may be associated with partial changes in acetylation degree [38], [39]. In contrast, the remaining samples exhibited Amide II bands within the standard range, indicating preservation of the chitin structure [38], [40]. The ChCl-Gly- NB 300 W sample also showed an Amide II peak at 1551 cm⁻^1^; however, differences in peak intensity compared with other samples may indicate more effective protein removal or structural modifications during extraction [41]. The chemically extracted sample (Chem) displayed all characteristic chitin peaks, including Amide I (1656 cm⁻^1^), Amide II (1551 cm⁻^1^), Amide III (1320 cm⁻^1^), and the β-glycosidic linkage (897 cm⁻^1^), although slight peak shifts relative to reference values were observed, possibly due to structural alterations caused by harsh acid–alkali treatment [41]. The presence of β-glycosidic bands within the range of 893–897 cm⁻^1^ in all samples confirmed preservation of the α-chitin structure [23]. Overall, the FTIR results demonstrated successful extraction of α-chitin with preserved characteristic functional groups. The degree of acetylation (DA) values calculated from FTIR spectra are summarized in (Table 3). The DA values for ChCl-Gly, ChCl-Gly-NB 100 W, ChCl-Gly-NB 200 W, ChCl-Gly-NB 300 W, and ChCl-Gly-NB 400 W were 80.1%, 72.4%, 74.8%, 78.6%, and 83.2%, respectively; in contrast, the chemically extracted sample (Chem) exhibited a DA value of 65.4%. All DES-NB samples maintained DA values above 70%, indicating substantial preservation of acetyl groups and the native chitin structure. The lower DA values observed in the chemically extracted sample may be attributed to harsher extraction conditions, which promote removal of acetyl groups from the polymer chain [40], [42], [43]. Among the advantages of using nanobubbles is the mild stimulation of the raw material without causing damage to the structure of target compounds [19]. These findings indicate that the presence of nanobubbles and lower temperature in the DES extraction process prevents excessive deacetylation and preserves the chitin structure, whereas in the chemical method, more extensive deacetylation occurs, which can be attributed to structural damage caused by harsh acid and alkali treatments and heat, leading to removal of more acetyl groups (–COCH_3_) from the polymer chain [40], [42], [43].

**Table 4:** Characteristic FTIR absorption bands of extracted chitin samples (cm⁻^1^).

**Band**	Vibrational assignment	**Chem**	**ChCl-Gly**	**ChCl-Gly- NB 100 W**	**ChCl-Gly- NB 200 W**	**ChCl-Gly- NB 300 W**	**ChCl-Gly- NB 400 W**
Amide A	N–H stretching	3448	3440	3438	3441	3502	3422
C–H	C–H stretching	2927	2925	2926	2927	2926	2925
Amide I	C=O stretching (amide carbonyl)	1656	1656	1655	1655	1656	1655
Amide II	N–H bending + C–N stretching	1551	1550	1530	1551	1551	1550
CH_3_ bending	Symmetric deformation of CH_3_	1378	1377	1378	1378	1377	1377
Amide III	C–N stretching + N–H deformation	1320	1316	1315	1316	1318	1315
β-glycosidic	C–O–C stretching	897	895	896	896	896	893

### 3.6 XRD analysis

**Fig. 2:**
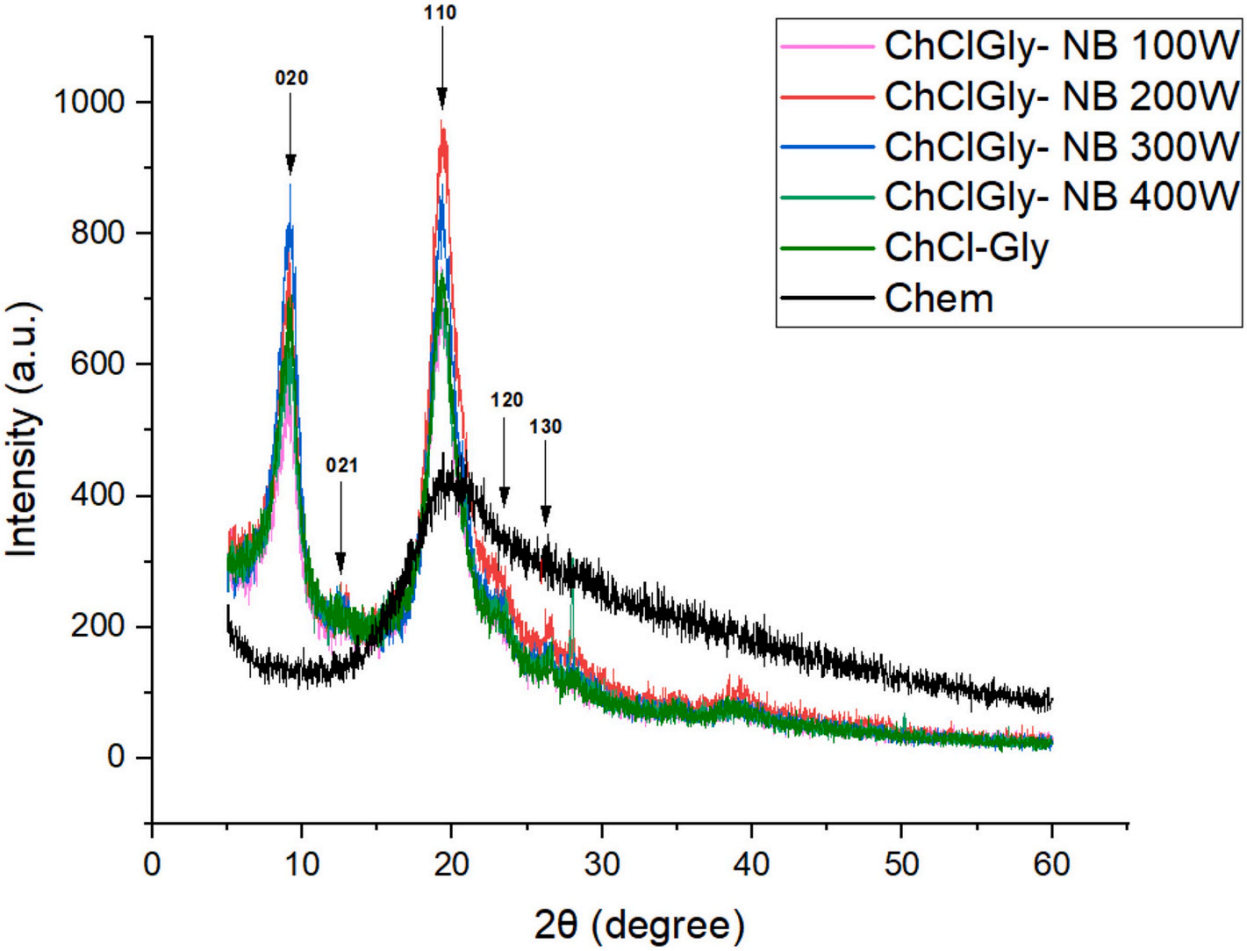
X-ray diffraction (XRD) analysis of all extracted chitin samples.

X-ray diffraction (XRD) analysis was performed to evaluate the crystalline structure and crystallinity index (CrI) of the extracted chitin samples. Crystallinity indices were calculated using the Segal method [24]. All extracted chitin samples exhibited the characteristic diffraction peaks of α-chitin, including the (020) reflection at approximately 9°, the (110) reflection at approximately 19°, and the weaker reflections corresponding to the (120) and (130) planes in the 23–26° range, which are consistent with the reported crystalline structure of α-chitin. (Fig. 2) [44] However, the chemically extracted sample lacked the characteristic (020) diffraction peak. The disappearance of the (020) peak in the chemical treatment may be associated with extensive deacetylation, as the intensity of this plane is strongly related to the presence of N-acetylated regions within the crystalline structure [45]. The consistency between FTIR and XRD analyses confirms the reliability of the structural characterization results. The highest crystallinity index was observed for the chemically extracted sample (91.30%), likely due to the removal of amorphous regions by strong acid treatment [23]. Among the DES-NB treatments, ChCl-Gly- NB 200 W exhibited the highest CrI value (82.1%), while the remaining samples showed CrI values ranging from 77.32% to 81.59%, which are close to the results reported by [35] with a crystallinity of 84%. Although the DES-NB samples exhibited slightly lower crystallinity compared with the chemical method, no impurity-related peaks associated with calcium carbonate or calcium phosphate were detected, indicating high phase purity of the extracted chitin [46].

### 3.7 TGA/DTG analysis

**Fig. 3:**
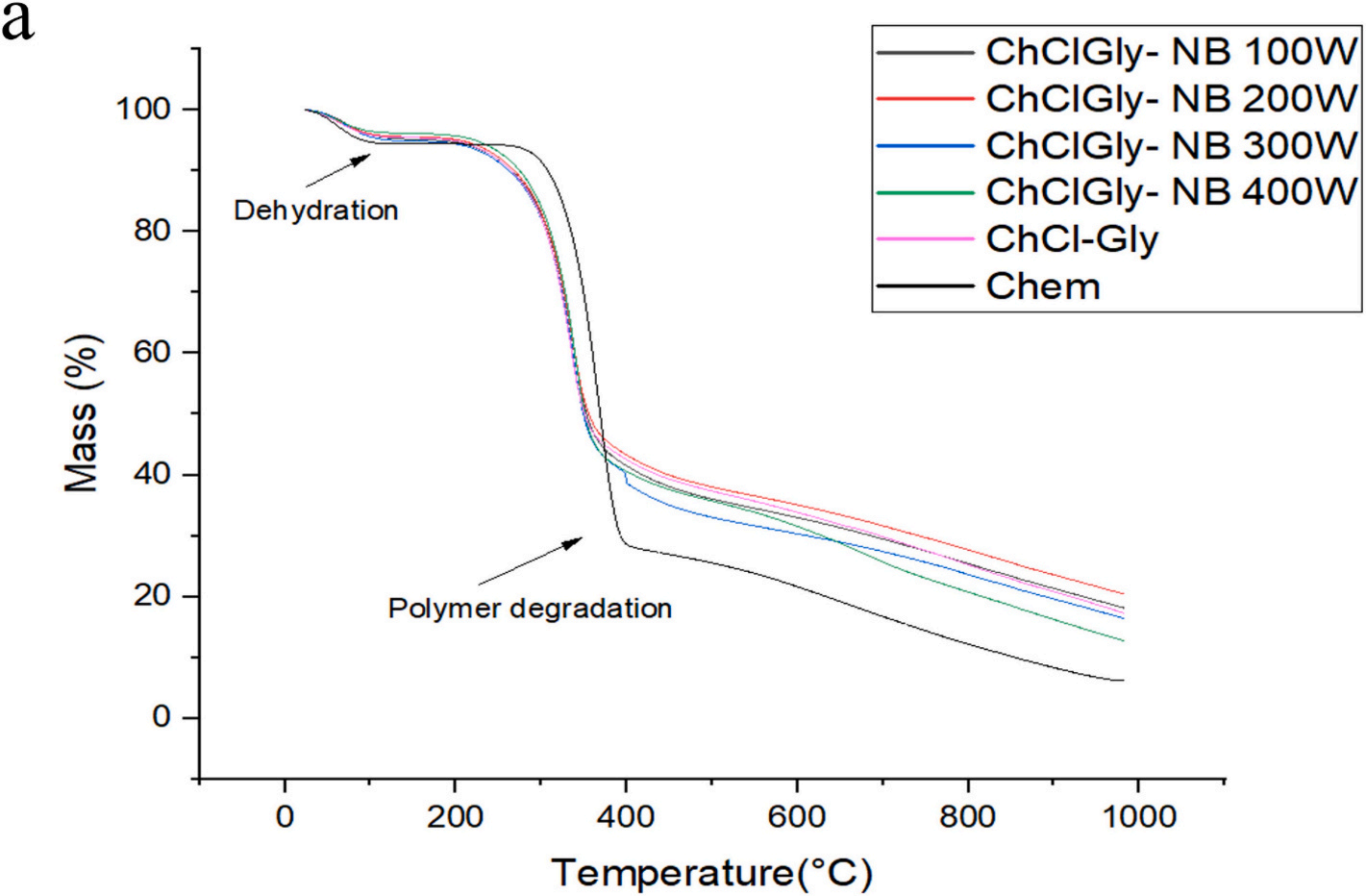

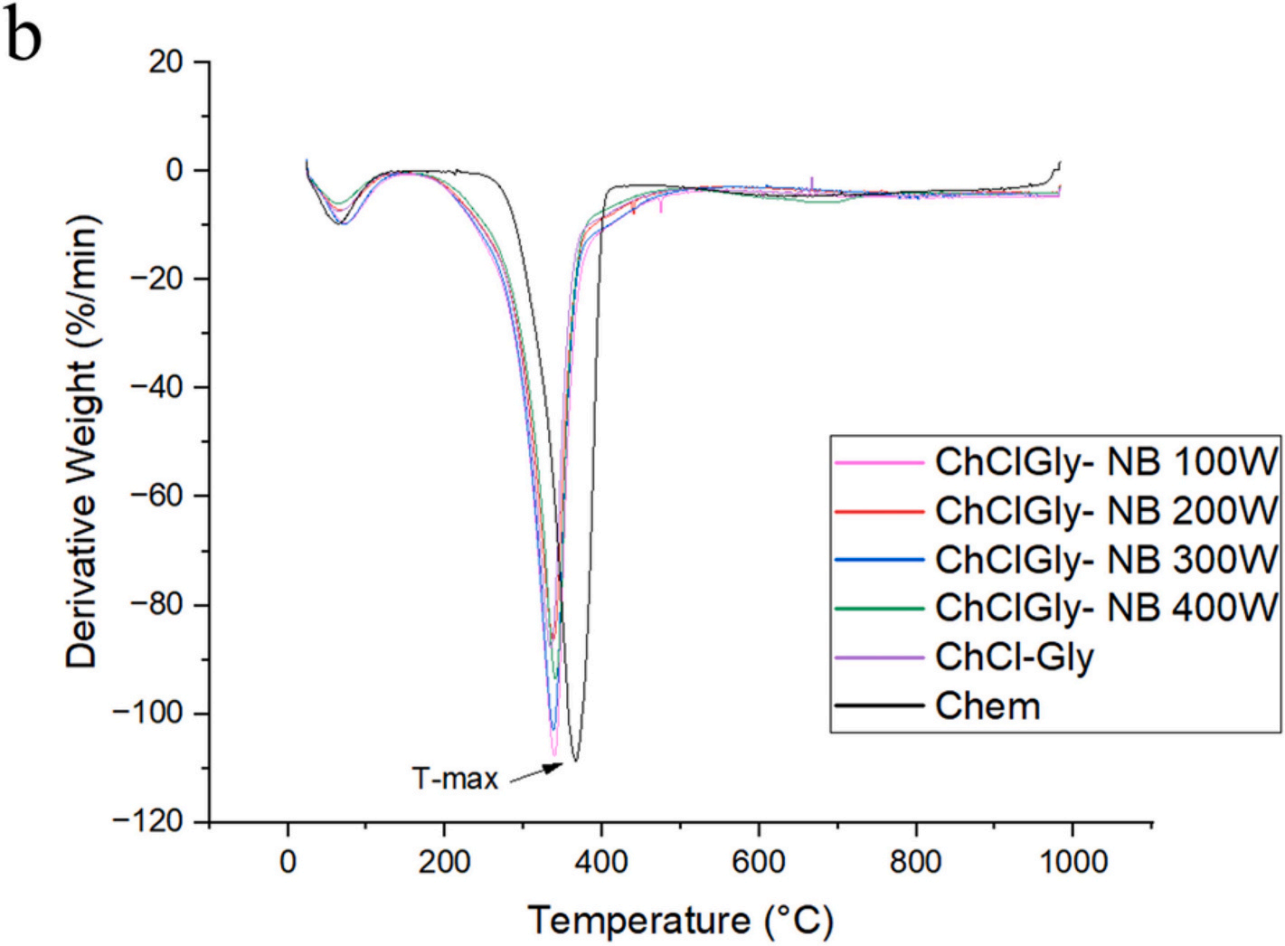
TGA (a) and DTG (b) curves of all extracted chitin samples.

The thermal stability of the extracted chitin samples was evaluated using TGA/DTG analysis under a nitrogen atmosphere over the temperature range of 30–1000 °C and the results are presented in Fig. 3 and Table 5. All thermograms exhibited three major degradation stages. The first stage (20–120 °C) with weight losses of approximately 3.7–5.1% corresponded to moisture evaporation. The second stage (230–410 °C), associated with polymer chain degradation, showed the highest mass loss (54–72%). The third stage (600–750 °C) was attributed to decomposition of residual calcium carbonate [47], [48]. The T_max_ values of DES-NB samples ranged from 334 to 339 °C, whereas the chemically extracted sample exhibited a substantially higher T _max_ value (368 °C) along with a greater second-stage mass loss (71.9%), indicating higher thermal stability. Samples with higher crystallinity generally exhibit enhanced thermal resistance [49], which is consistent with the FTIR and XRD results obtained in this study. The chemically extracted sample also exhibited the lowest residual weight (6.6%), compared with 16–21% for DES-treated samples, indicating lower ash content and more extensive mineral removal. These observations are in agreement with the demineralization results, which confirmed the superior mineral removal efficiency of strong acid treatment.

**Table 5:** Thermal degradation parameters of extracted chitin samples.

**Treatment**	**First-stage of weight loss** (**%**)	**Second-stage of weight loss** (**%**)	**residual weight** (**%**)	**T _max_** (**°C**)
**ChCl-Gly - NB 100 W**	3.9	56.9	18.8	339
**ChCl-Gly- NB 200 W**	3.8	54.1	21.1	337
**ChCl-Gly- NB 300 W**	3.7	55.2	16.9	339
**ChCl-Gly- NB 400 W**	3.7	55.1	18	336
**ChCl-Gly**	3.9	54	18.8	334
**Chem**	5.1	71.9	6.6	368

### 3.8 SEM analysis

**Fig. 4:**
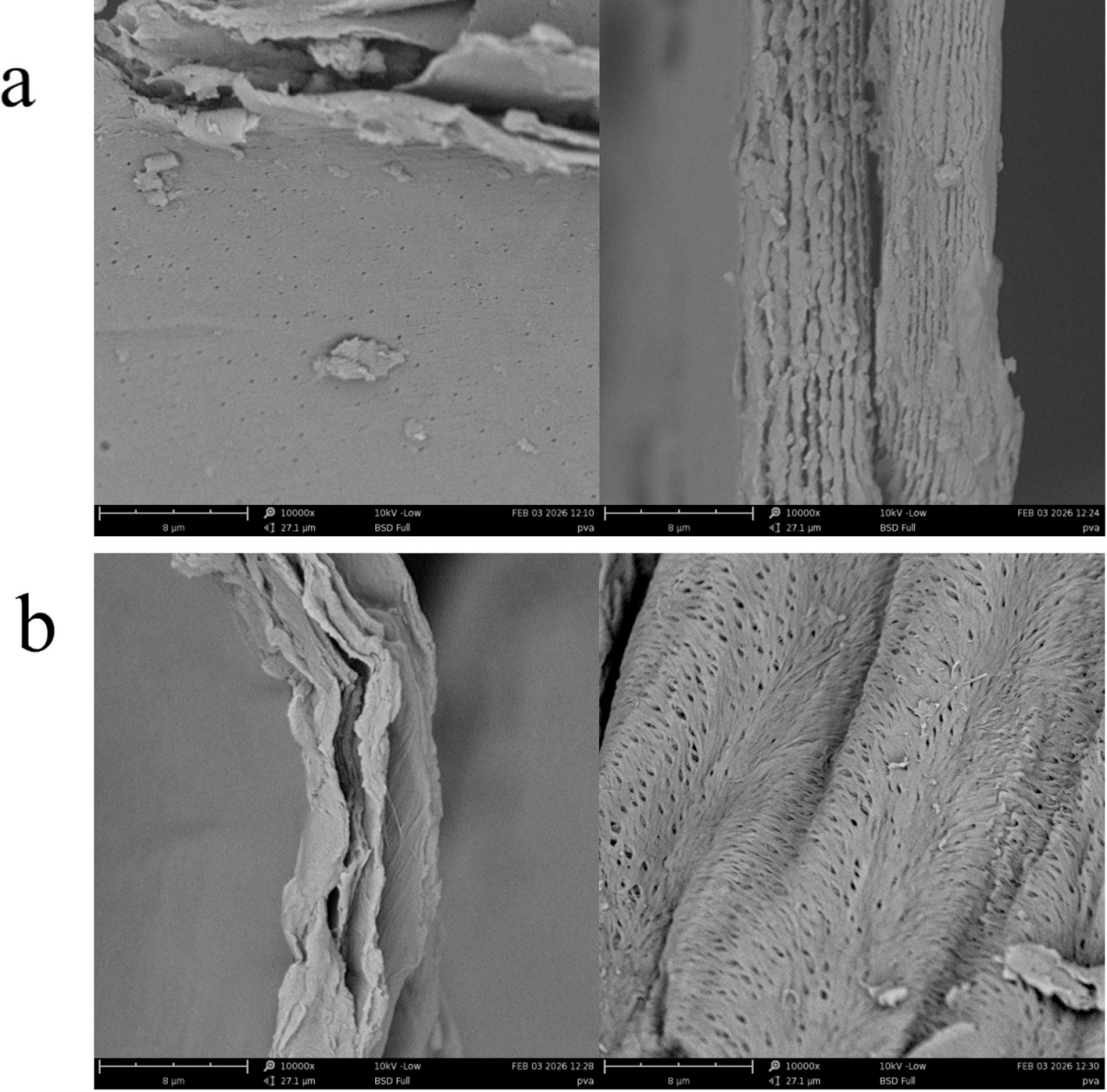
SEM image of (a) DES-NB-400 W-extracted chitin samples and (b) chemically extracted chitin samples (Magnification: 8000×).

Scanning electron microscopy (SEM) was used to examine the surface morphology of the extracted chitin samples (Fig. 4). All samples, including those obtained through conventional chemical extraction and DES-based extraction assisted by ultrasound-generated nanobubbles, exhibited a well-developed porous and fibrous morphology. This morphology is characteristic of the chitin fibrillar network and indicates the effective removal of non-chitinous components, particularly proteins and mineral matter, from the shrimp shell matrix [16], [21]. Residual proteins and calcium carbonate can cover the chitin fibrils and obscure their intrinsic morphology; therefore, the clear exposure of the fibrous network observed in all samples provides morphological evidence of efficient purification [50]. The DES–NB treatments exhibited a distinctly developed porous–fibrous architecture, which may be attributed to the combined effects of the DES extraction medium and nanobubble-induced cavitation. The collapse of nanobubbles can generate localized shear forces and microjets, promoting disruption of the shell matrix, facilitating the release of entrapped proteins and minerals, and enhancing mass transfer between the extraction medium and the shrimp shell matrix [31]. These effects may contribute to the formation of the porous morphology observed after extraction. The porous–fibrous structure is also of functional importance because it provides greater surface accessibility and exposes a larger number of chitin functional groups. Consequently, the extracted chitin may possess favorable structural characteristics for potential applications in areas such as pollutant adsorption, tissue engineering, and drug delivery. Overall, the SEM results, together with the FTIR, XRD, and compositional analyses, support the successful removal of non-chitinous components and demonstrate that the DES–NB strategy can produce chitin with a well-developed porous fibrous microstructure.

## 4 Conclusion

A green and efficient strategy for chitin extraction from *L. vannamei* shell waste was successfully developed using a ChCl-Gly deep eutectic solvent coupled with ultrasound-assisted nanobubbles. Compared with the conventional chemical method, the DES-NB system significantly enhanced chitin recovery, achieving a maximum extraction yield of 41.40% versus 17.91% for chemical extraction. Nanobubble-assisted treatments also improved deproteinization and demineralization efficiencies, reaching values above 97% and 90%, respectively. Among the investigated treatments, ChCl-Gly- NB 400 W provided the best overall balance between extraction efficiency, structural preservation, crystallinity, and thermal stability. FTIR and XRD analyses confirmed the preservation of the characteristic α-chitin structure, while SEM micrographs revealed a porous fibrous morphology associated with effective impurity removal. In addition, the DES-NB system enabled efficient extraction under relatively mild conditions without the extensive use of concentrated acids and alkalis. The enhanced extraction performance was mainly attributed to cavitation-induced nanobubble effects, which promoted mass transfer and facilitated impurity removal from the shrimp shell matrix. Overall, the proposed DES-NB approach represents a sustainable alternative for chitin extraction and shell waste valorization, with strong potential for future industrial and biorefinery applications.

## CRediT authorship contribution statement

**Azadeh Ghiasvand:** Writing – original draft, Methodology, Investigation, Data curation. **Masoud Rezaei:** Writing – review & editing, Visualization, Supervision, Funding acquisition, Formal analysis, Conceptualization. **Shahab Naghdi:** Writing – review & editing, Methodology, Conceptualization.

## Funding

This research was funded by the Iran National Science Foundation (INSF, Grant Number: 4033185).

## Declaration of competing interest

The authors declare that they have no known competing financial interests or personal relationships that could have appeared to influence the work reported in this paper.

## References

[1] Nirmal N.P., Santivarangkna C., Rajput M.S., Benjakul S. (2020). Trends in shrimp processing waste utilization: an industrial prospective. Trends Food Sci. Technol..

[2] M. Djellouli, M.Y. Arancibia, Antioxidant and antimicrobial enhancement by reaction of protein hydrolysates derived from shrimp by- products with glucosamine, (2015) 1–36.

[3] Priyangga A., Atmaja L., Santoso M., Jaafar J., Santoso E., Hartanto D., Adya Salsabila R., Ningtyas A., Putra Ramdhani E. (2023). The potential development of shrimp shell waste into chitosan originating from Pacitan Coast, Indonesia. BIO Web Conf..

[4] Z. Liu, Q. Liu, D. Zhang, S. Wei, Q. Sun, Q. Xia, W. Shi, H. Ji, S. Liu, Comparison of the Proximate Composition and Nutritional Profile of Byproducts and Edible Parts of Five Species of Shrimp, (2021) 1–16. 10.3390/foods10112603 PMC8619515 34828883

[5] Sulthan R., Reghunadhan A., Sambhudevan S. (2023). A new era of chitin synthesis and dissolution using deep eutectic solvents-comparison with ionic liquids. J. Mol. Liq..

[6] Nag S., Das Saha K. (2021). Chitosan-decorated PLGA-NPs loaded with tannic acid/vitamin E mitigate colon cancer via the NF-κB/β-Cat/EMT pathway. ACS Omega.

[7] Harugade A., Sherje A.P., Pethe A. (2023). Chitosan : a review on properties, biological activities and recent progress in biomedical application. React. Funct. Polym..

[8] Hajji S., Ghorbel-Bellaaj O., Younes I., Jellouli K., Nasri M. (2015). Chitin extraction from crab shells by bacillus bacteria. Biological activities of fermented crab supernatants. Int. J. Biol. Macromol..

[9] Kadokawa J.I., Setoguchi T., Yamamoto K. (2013). Preparation of highly flexible chitin nanofiber-graft-poly(γ-l- glutamic acid) network film. Polym. Bull..

[10] Negi T., Kumar A., Sharma S.K., Rawat N., Saini D., Sirohi R., Prakash O., Dubey A., Dutta A., Shahi N.C. (2024). Deep eutectic solvents: preparation, properties, and food applications. Heliyon.

[11] Zdanowicz M., Wilpiszewska K., Spychaj T. (2018). Deep eutectic solvents for polysaccharides processing. a review. Carbohydr. Polym..

[12] Huang H., Ying P., Wang Y., Wu Q., Wang L., Fu X. (2025). Temperature-dependent convection induced incremental extraction of anthocyanins from Melastoma dodecandrum Lour. Based on recyclable natural deep eutectic system. Food Chem..

[13] Huang H., Guo S., Xu Y., Ettoumi F., Fang J., Yan X., Xie Z., Luo Z., Cheng K. (2024). Valorization and protection of anthocyanins from strawberries (Fragaria× ananassa Duch.) by acidified natural deep eutectic solvent based on intermolecular interaction. Food Chem..

[14] Khajavian M., Vatanpour V., Castro-Muñoz R., Boczkaj G. (2022). Chitin and derivative chitosan-based structures—preparation strategies aided by deep eutectic solvents: a review. Carbohydr. Polym..

[15] Li R., Hsueh P.H., Ulfadillah S.A., Wang S.T., Tsai M.L. (2024). Exploring the sustainable utilization of deep eutectic solvents for chitin isolation from diverse sources. Polymers (Basel).

[16] Zhao D., Huang W.C., Guo N., Zhang S., Xue C., Mao X. (2019). Two-step separation of chitin from shrimp shells using citric acid and deep eutectic solvents with the assistance of microwave. Polymers (Basel).

[17] Sharma M., Mukesh C., Mondal D., Prasad K. (2013). Dissolution of α-chitin in deep eutectic solvents. RSC Adv..

[18] Nirmalkar N., Pacek A.W., Barigou M. (2018). On the existence and stability of bulk nanobubbles. Langmuir.

[19] Uchida T., Liu S., Enari M., Oshita S., Yamazaki K., Gohara K. (2016). Effect of NaCl on the lifetime of micro- and nanobubbles. Nanomaterials.

[20] Said Al Hoqani H.A., AL-Shaqsi N., Hossain M.A., Al Sibani M.A. (2020). Isolation and optimization of the method for industrial production of chitin and chitosan from Omani shrimp shell. Carbohydr. Res..

[21] Zhang J., Xu W.R., Zhang Y.C. (2022). Facile production of chitin from shrimp shells using a deep eutectic solvent and acetic acid. RSC Adv..

[22] Nirmalkar N., Pacek A.W., Barigou M. (2019). Bulk nanobubbles from acoustically cavitated aqueous organic solvent mixtures. Langmuir.

[23] Rahayu A.P., Islami A.F., Saputra E., Sulmartiwi L., Rahmah A.U., Kurnia K.A. (2022). The impact of the different types of acid solution on the extraction and adsorption performance of chitin from shrimp shell waste. Int. J. Biol. Macromol..

[24] Sun X., Wei Q., Yang Y., Xiao Z., Ren X. (2022). In-depth study on the extraction and mechanism of high-purity chitin based on NADESs method. J. Environ. Chem. Eng..

[25] Chauhan V., Ahalavat S., Singh A., Singh A., Verma S. (2025). Characterization of biscuits fortified with shrimp (Litopenaeus vannamei). Waste Protein Concentrate.

[26] Rødde R.H., Einbu A., Vårum K.M. (2008). A seasonal study of the chemical composition and chitin quality of shrimp shells obtained from northern shrimp (Pandalus borealis). Carbohydr. Polym..

[27] Saravana P.S., Ho T.C., Chae S.-J., Cho Y.-J., Park J.-S., Lee H.-J., Chun B.-S. (2018). Deep eutectic solvent-based extraction and fabrication of chitin films from crustacean waste. Carbohydr. Polym..

[28] Smith E.L., Abbott A.P., Ryder K.S. (2014). Deep eutectic solvents (DESs) and their applications. Chem. Rev..

[29] Dai Y., van Spronsen J., Witkamp G.J., Verpoorte R., Choi Y.H. (2013). Natural deep eutectic solvents as new potential media for green technology. Anal. Chim. Acta.

[30] Montazeri S.M., Kalogerakis N., Kolliopoulos G. (2023). Effect of chemical species and temperature on the stability of air nanobubbles. Sci. Rep..

[31] Javed M., Matloob A., Ettoumi F., Sheikh A.R., Zhang R., Xu Y. (2023). Novel nanobubble technology in food science: application and mechanism. Food Innovation Adv..

[32] Kasaai M.R. (2009). Various methods for determination of the degree of N-acetylation of chitin and chitosan: a review. J. Agric. Food Chem..

[33] Javed M., Belwal T., Ruyuan Z., Xu Y., Li L., Luo Z. (2022). Optimization and mechanism of phytochemicals extraction from camellia oleifera shells using novel biosurfactant nanobubbles solution coupled with ultrasonication. Food Bioproc. Tech..

[34] Ma J., Yu Y., Chu D., Zhu S., Liu Q., Yin H. (2024). Efficient and eco-friendly chitin production from crab shells using novel deep eutectic solvents. J. Polym. Environ..

[35] Zhang Q., Duan L., Li Y. (2022). Positive effects and mechanism of ultrasound on chitin preparation from shrimp shells by co-fermentation. Ultrason. Sonochem..

[36] S. Cho, J. Kim, J. Chun, J. Kim, Ultrasonic formation of nanobubbles and their zeta-potentials in aqueous electrolyte and surfactant solutions, 269 (2005) 28–34. https://doi.org/10.1016/j.colsurfa.2005.06.063.

[37] Javed M., Belwal T., Huang H., Xu Y., Ettoumi F., Li L., Fang X., Luo Z. (2022). Generation and stabilization of CO2 nanobubbles using surfactants for extraction of polyphenols from Camellia oleifera shells. J. Food Sci..

[38] J. Kumirska, M. Czerwicka, Z. Kaczyński, A. Bychowska, K. Brzozowski, J. Thöming, P. Stepnowski, Kumirska, J., et al., Application of spectroscopic methods for structural analysis of chitin and chitosan, Mar. Drugs 8(5) (2010) 1567–1636. 10.3390/md8051567 PMC2885081 20559489

[39] S.N. Ghanem, M.I. Marzouk, M.E. Tawfik, S.B. Eskander, Spectroscopic approaches for structural analysis of extracted chitosan generated from chitin deacetylated for escalated periods, (2025). 10.1186/s13065-025-01558-3 PMC12265149 40671155

[40] J. Desbrie, J. Brugnerotto, J. Lizardi, F.M. Goycoolea, W. Argu, An infrared investigation in relation with chitin and chitosan characterization, 42 (2001).

[41] Pereira A.G.B., Muniz E.C., Hsieh Y.-L. (2014). Chitosan-sheath and chitin-core nanowhiskers. Carbohydr. Polym..

[42] Atmaja L., Manimoy H., Arizka L.E. (2019). Modification of chitosan-chitosan phtalate anhydrides matrices. IPTEK the J. Technol. Sci..

[43] Shigemasa Y., Matsuura H., Sashiwa H., Saimoto H. (1996). Evaluation of different absorbance ratios from infrared spectroscopy for analyzing the degree of deacetylation in chitin. Int. J. Biol. Macromol..

[44] Goodrich J.D., Winter W.T. (2007). α-Chitin nanocrystals prepared from shrimp shells and their specific surface area measurement. Biomacromolecules.

[45] Zhang Y., Xue C., Xue Y., Gao R., Zhang X. (2005). Determination of the degree of deacetylation of chitin and chitosan by X-ray powder diffraction. Carbohydr. Res..

[46] Hai T.C., Dat N.T., Tuan Q.L.A., Ngan H.L.T., Tu N.D.T., Thinh N.T., Quang D.N., Anh L.T.H., Van Man P. (2024). Extraction of chitin from giant tiger prawn (Penaeus monodon) shrimp shell using deep eutectic solvents and citric acid. Appl. Sci. Eng. Prog..

[47] Paulino A.T., Simionato J.I., Garcia J.C., Nozaki J. (2006). Characterization of chitosan and chitin produced from silkworm crysalides. Carbohydr. Polym..

[48] Li F., You X., Li Q., Qin D., Wang M., Yuan S., Chen X., Bi S. (2021). International Journal of Biological Macromolecules Homogeneous deacetylation and degradation of chitin in NaOH / urea dissolution system. Int. J. Biol. Macromol..

[49] R. Amin, M.A. Chowdhury, Heliyon Characterization and performance analysis of composite bioplastics synthesized using titanium dioxide nanoparticles with corn starch, 5 (2019). https://doi.org/10.1016/j.heliyon.2019.e02009. 10.1016/j.heliyon.2019.e02009 PMC6722259 31497660

[50] Cui D., Yang J., Lu B., Deng L., Shen H. (2022). International Journal of Biological Macromolecules Extraction and characterization of chitin from Oratosquilla oratoria shell waste and its application in *Brassica campestris* L . ssp. Int. J. Biol. Macromol..

